# Transcriptional Profiling of Normal, Stenotic, and Regurgitant Human Aortic Valves

**DOI:** 10.3390/genes11070789

**Published:** 2020-07-14

**Authors:** Christina L. Greene, Kevin J. Jaatinen, Hanjay Wang, Tiffany K. Koyano, Mary S. Bilbao, Y. Joseph Woo

**Affiliations:** 1 Department of Cardiothoracic Surgery, Stanford University, Stanford, CA 94305, USA; christina.greene@cardio.chboston.org (C.L.G.); kevinjaatinen@gmail.com (K.J.J.); hanjay@stanford.edu (H.W.); tkoyano3@stanford.edu (T.K.K.); msbilbao@stanfordhealthcare.org (M.S.B.); 2 Stanford Cardiovascular Institute, Stanford University, Stanford, CA 94305, USA; 3 Department of Bioengineering, Stanford University, Stanford, CA 94305, USA

**Keywords:** aortic stenosis, aortic insufficiency, transcriptional profiling, RNA sequencing

## Abstract

The genetic mechanisms underlying aortic stenosis (AS) and aortic insufficiency (AI) disease progression remain unclear. We hypothesized that normal aortic valves and those with AS or AI all exhibit unique transcriptional profiles. Normal control (NC) aortic valves were collected from non-matched donor hearts that were otherwise acceptable for transplantation (*n* = 5). Valves with AS or AI (*n* = 5, each) were collected from patients undergoing surgical aortic valve replacement. High-throughput sequencing of total RNA revealed 6438 differentially expressed genes (DEGs) for AS vs. NC, 4994 DEGs for AI vs. NC, and 2771 DEGs for AS vs. AI. Among 21 DEGs of interest, *APCDD1L*, *CDH6*, *COL10A1*, *HBB*, *IBSP*, *KRT14*, *PLEKHS1*, *PRSS35*, and *TDO2* were upregulated in both AS and AI compared to NC, whereas *ALDH1L1*, *EPHB1*, *GPX3*, *HIF3A*, and *KCNT1* were downregulated in both AS and AI (*p* < 0.05). *COL11A1*, *H19*, *HIF1A*, *KCNJ6*, *PRND*, and *SPP1* were upregulated only in AS, and *NPY* was downregulated only in AS (*p* < 0.05). The functional network for AS clustered around ion regulation, immune regulation, and lipid homeostasis, and that for AI clustered around ERK1/2 regulation. Overall, we report transcriptional profiling data for normal human aortic valves from non-matched donor hearts that were acceptable for transplantation and demonstrated that valves with AS and AI possess unique genetic signatures. These data create a roadmap for the development of novel therapeutics to treat AS and AI.

## 1. Introduction

Valvular heart disease is increasingly being recognized as a major contributor to cardiovascular morbidity and mortality [1,2]. Globally, 31 million people are affected by non-rheumatic valvular disease, and the number of associated deaths has increased by 112% since 1990, due to aging and population growth around the world [3]. Indeed, in the United States, the prevalence of valvular heart disease is particularly high among the elderly, including 8% of those aged 65 years and older, and 13% of those aged 75 years and older [4]. Thus, given our rapidly aging global society, valvular heart disease represents an underappreciated yet serious looming public health problem.

Aortic valve disease is a common type of valvular heart disease and may be categorized as aortic stenosis (AS) or aortic insufficiency (AI). AS is characterized by rigid, thickened, and calcified leaflets, which impede the flow of blood out of the heart [5]. Hemodynamic stress, which may be induced by inflammation, rheumatic disease, congenital bicuspid valve deformities, or other etiologies, results in activation of various cell signaling pathways that are thought to promote maladaptive leaflet remodeling and valve ossification [6,7]. To compensate for increased afterload, the left ventricle gradually hypertrophies but ultimately fails, often leading to death within 3 years of symptom onset unless the valve is replaced [5,8,9,10]. In contrast, AI develops when the aortic valve leaflets fail to coapt properly, often due to primary dilation of the aortic root, annulus, or ascending aorta, or due to rheumatic disease, congenital bicuspid valve disease, or other etiologies [11]. As blood refluxes into the heart during each diastolic cycle, the left ventricle eccentrically hypertrophies and dilates, ultimately resulting in heart failure with 10% mortality per year among symptomatic patients [12].

In both cases, AS and AI require decades of gradual disease progression before symptoms develop, at which point the prognosis sharply declines [5,8,12]. This long latency period lends itself to possible therapeutic interventions aimed at reversing disease progression and potentially avoiding future surgical and transcatheter valve replacement procedures. However, the development of novel therapeutic options for aortic valve disease has been inhibited by an inadequate understanding of the genetic and molecular mechanisms underlying AS and AI disease progression. Although RNA profiling has previously been performed for human aortic valves with AS [13,14,15,16], to our knowledge, no study has examined the RNA profile of human aortic valves with AI. Furthermore, these previous studies utilized aortic valves from failing hearts explanted during transplant or aortic valves excised during aortic root aneurysm surgery as “normal” controls, although a clear cardiac disease process was involved in all cases.

Here, we present the transcriptional profile of human aortic valves with AS or AI in comparison to normal human aortic valves from non-matched donor hearts that were acceptable for transplantation. We hypothesized that there exist unique, key regulatory pathways governing the pathobiology of AS and AI, both of which may be distinguished from the baseline homeostatic profile of normal aortic valves.

## 2. Materials and Methods

### 2.1. Selection of Normal Control and Diseased Valves

Normal control (NC) aortic valves were collected from donor hearts (*n* = 5) that were acceptable for transplantation, but which were not matched with a suitable recipient. These unmatched donor hearts were identified by our cardiothoracic surgery transplant team and our local organ procurement organization. The inclusion criteria for the NC group included a maximum travel distance of 60 miles or maximum travel time of 90 min, total ischemic time under 6 h, no valvular disease on echocardiography, no positive blood cultures, no history of human immunodeficiency virus infection, no autoimmune disease, no blunt chest trauma, and confirmed consent for research, which was obtained from the family representing the donor by our local organ procurement organization in contract with Stanford University. Once the heart arrived at our laboratory, the aortic valve leaflets were excised, flash frozen, and stored at −80 °C until use.

Leaflet tissue from diseased aortic valves was collected from patients undergoing elective aortic valve replacement at our hospital. The excised tissues were flash frozen and stored at −80 °C until use. Valves included in the AS group (*n* = 5) were required to have severe disease defined by a mean aortic valve gradient >40 mmHg and aortic valve area <1.0 cm^2^. Valves included in the AI group (*n* = 5) were required to have moderate or severe disease defined qualitatively on echocardiography. Valves from patients with infection, autoimmune disease, or connective tissue disease were excluded.

Our study was approved by the Institutional Review Board of Stanford University (IRB 32769 and 40566). All patients consented for participation in the study.

### 2.2. RNA Extraction and Sequencing

Total RNA was extracted from whole aortic valve leaflets using the PureLink™ RNA Minikit (Thermo Fisher Scientific, Waltham, MA, USA) with on-column DNase digestion according to the manufacturer’s instructions. A 10-min proteinase K digestion was added during the lysis step to further homogenize the tissue. RNA integrity was assessed with an Agilent 2100 Bioanalyzer (Agilent, Santa Clara, CA, USA). RNA integrity scores were between 7.4 and 9.2. A complementary DNA (cDNA) strand-specific library was created in duplicate using NEBNext^®^ Ultra™ Directional RNA Library Prep Kit for Illumina^®^ (New England Biolabs Inc., Ipswich, MA, USA). After library construction, samples were diluted and quantified by Qubit^®^ 2.0 Fluorometer (Life Technologies, Carlsbad, CA, USA). cDNA insert size was detected using an Agilent 2100 Bioanalyzer. Library quality was assessed by quantitative polymerase chain reaction (qPCR) ensuring effective concentration >2 nM. cDNA libraries were sequenced using Illumina HiSeq 2500 (Illumina^®^, San Diego, CA, USA). The 150-bp paired-ends reads were generated with approximately 30 million reads per sample. Sequencing was performed in duplicate for each sample. The RNA sequencing data for this study can be acquired from the National Center for Biotechnology Information’s Gene Expression Omnibus (accession number GSE153555).

### 2.3. RNA Sequencing Analysis

The raw sequencing reads were cleaned by removing low-quality reads, adapters, and reads with >10% indeterminable bases. The Q20 scores, Q30 scores, and guanine-cytosine content were calculated. The reference genome and gene model annotation files were downloaded from human genome websites (http://www.ncbi.nlm.nih.gov, http://uswest.ensembl.org/Homo_sapiens, http://genome.ucsc.edu). Indexes of the reference genome were built using Bowtie v2.0.6 [17]. Paired-end clean reads were aligned to the reference genome using Spliced Transcripts Alignment to a Reference (STAR) software v2.5 [18]. HTSeq v0.6.1 was used to count the read numbers mapped to each gene [19]. Fragments per kilobases per million (FPKM) were calculated to estimate the level of gene expression [20]. Pearson R^2^ correlation coefficients were calculated between samples. Differential gene expression analysis was performed by inputting normalized read counts into the DESeq2 R package [21]. A model based on the negative binomial distribution was used to determine differential gene expression (log_2_(fold change)). *p*-values were adjusted using the Benjamini-Hochberg approach for controlling false discovery rate [22]. Genes with adjusted *p*-values < 0.05 found by DESeq2 were assigned as differentially expressed genes (DEGs). Cluster analysis of DEGs was performed using log_10_(FPKM + 1) of the union of DEGs of AS, AI, and NC valves. Hierarchal clustering distance was expressed as a heat map with self-organizing mapping and k-means using silhouette coefficient to adapt the optimal classification with default parameters in R.

### 2.4. Quantitative Polymerase Chain Reaction

qPCR was performed to confirm the expression of genes identified by RNA sequencing. RNA was extracted as described above. A cDNA library was created from each sample using iScript™ cDNA Synthesis Kit (BIO-RAD, Hercules, CA, USA). Libraries were diluted 10:1 prior to qPCR analysis. TaqMan Gene Expression Assays (Applied Biosystems, Foster City, CA, USA) with FAM™ fluorescent dye reporters were used to quantify expression of our genes of interest (GOI) according to the manufacturer’s instructions. These reactions were multiplexed with the housekeeping gene 18S^®^ TaqMan^®^ Gene Expression Assay with VIC^®^ fluorescent dye reporter to generate cycle threshold (CT) values to calculate relative gene expression using the comparative CT method. The standard protocol for TaqMan^®^ technologies qPCR was performed using the Viia 7 Real-Time PCR System. ΔCT values were calculated for every sample as CT_GOI_-CT_18S_. The average ΔCT value was calculated for the NC group. ΔΔCT values were calculated for each sample as ΔCT_sample_ − *Average*ΔCT_NC_. Finally, fold change was calculated as 2^−ΔΔCT^.

### 2.5. Computational Modeling

Gene ontology (GO) enrichment analysis was performed using GOseq R package and topGO [23], based on Wallenius non-central hyper-geometric distribution. GO terms with corrected *p*-values < 0.05 were considered as significantly enriched DEGs. Kyoto Encyclopedia of Genes and Genome (KEGG) pathway enrichment analysis was performed using KOBAS software to test the statistical enrichment of DEGs [24]. GO and KEGG analysis of DEGs was performed using the clusterProfiler R package [25]. Gene length bias was corrected, and enriched genes were considered significant by a *p*-value < 0.05. Cytoscape and the ClueGO plug-in were used to create a functional network [26,27], based on DEGs with an absolute fold change ≥3 for AS and ≥1.5 for AI. Only DEGs which were unique to AS or AI were included in the network (*n* = 3182 and *n* = 1738 respectively). GO terms relating to biological processes with a *p*-value < 0.05 after a Benjamini-Hochberg correction were mapped, with each node representing a GO term, a group of nodes defining a cluster, and interacting clusters defining a system. Clusters which represented the major connections between systems were designated as high-impact bridges. GO terms between levels 3 and 8 were used. A minimum of three genes and 4% of the associated genes for a specific GO term were required to represent a node on the functional network, based on predefined selection criteria of the ClueGo plugin. A kappa score >0.4 was used, which characterizes a GO term to GO term relationship and functional group in ClueGO such that nodes with less than moderate agreement are excluded [28].

A protein-protein interaction (PPI) network was created using the STRING Database [29]. The PPI network for differential gene expression was generated using NCBI BLAST v2.2.28 protein interaction database (http://string-db.org/). A STRING score of 0.7 was used as a cutoff for inclusion in the network.

### 2.6. Statistical Analysis

Statistical analysis was performed using GraphPad Prism version 7.00 (GraphPad Software, La Jolla, CA, USA). Data are reported as mean with standard error except where otherwise noted. Kruskal-Wallis one-way analysis of variance and the Mann-Whitney test were performed for statistical comparisons. Differences were considered statistically significant at *p*-values < 0.05.

## 3. Results

### 3.1. Baseline Patient Characteristics

A total of 15 patients were included in this study, with five patients in each of the NC, AS, and AI groups (Table 1). All patients in the AS group (age 73.8 ± 1.4 years old) exhibited severe AS, with four patients having a tri-leaflet aortic valve, and one patient having a bicuspid aortic valve. Among patients in the AI group (age 69.0 ± 4.5 years old), three had severe AI while two had moderate AI. All patients in the AI group had tri-leaflet aortic valves. None of the patients in the AS group exhibited worse than mild AI, and none of the patients in the AI group exhibited worse than mild AS. None of the AS or AI patients had rheumatic valve disease. All patients in the NC group (age 37.2 ± 9.0 years old) had no remarkable cardiac or valvular disease.

### 3.2. Results of RNA Sequencing and Quantitative Polymerase Chain Reaction

**Figure 1:**
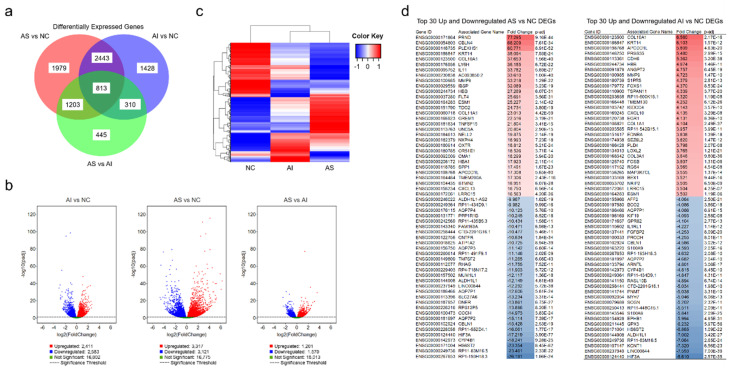
Differentially expressed genes in aortic valves after RNA Sequencing. (**a**) Venn diagram of differentially expressed genes (DEGs) in valves with aortic stenosis (AS), aortic insufficiency (AI), and normal controls (NC). (**b**) Volcano plots representing the distribution of gene expression in AS, AI, and NC aortic valves. The gene expression of AI vs. NC, AS vs. NC, and AS vs. AI are represented relative to statistical significance values (*p* < 0.05). The *x*-axis represents relative fold change, while the *y*-axis represents statistical significance. Each point represents a gene and its expression. (**c**) Cluster analysis of DEGs in the AS, AI, and NC groups. Increased expression is represented in red while decreased expression is represented in blue. (**d**) The top 30 upregulated and downregulated genes identified by RNA sequencing are identified for AS vs. NC and AI vs. NC. Gene identification and gene names are listed with the fold change and adjusted *p*-value (*p*-adj). Red represents upregulated genes relative to NC and blue represents downregulated genes relative to NC.

RNA sequencing analysis identified 8621 DEGs among the AS, AI, and NC valves. Specifically, 6438 genes were found to be differentially expressed between the AS and NC groups; 4994 genes were found to be differentially expressed between the AI and NC groups; and 2771 genes were found to be differentially expressed between the AS and AI groups. Of these, 1979 (31%) genes were expressed at statistically different levels between the AS and NC groups, and 1428 (29%) genes were expressed at statistically different levels between the AI and NC groups (*p* < 0.05, Figure 1a). The most prominent differences were observed between the AS and NC groups (Figure 1b), and cluster analysis showed that genetic expression between the AS and NC groups appeared to be inverted (Figure 1c). The top 30 upregulated and downregulated DEGs among the AS versus NC groups and the AI versus NC groups are listed in Figure 1d.

Genetic expression was confirmed using both RNA sequencing and qPCR for 21 DEGs of interest, which either exhibited the greatest expression differences between the AS or AI groups compared to the NC group, or were previously identified as DEGs in prior studies (Table 2). Specifically, based on RNA sequencing results, the genes *APCDD1L*, *CDH6*, *COL10A1*, *IBSP*, *KRT14*, *PLEKHS1*, *PRSS35*, and *TDO2* were significantly upregulated in both AS and AI valves compared to NC valves, while *ALDH1L1*, *EPHB1*, *GPX3*, *HIF3A*, and *KCNT1* were significantly downregulated in both AS and AI valves compared to NC valves. Genes that were significantly upregulated only in AS versus NC valves included *COL11A1*, *H19*, *HBB*, *HIF1A*, *KCNJ6*, *PRND*, and *SPP1*, while *NPY* was the only gene found to specifically be downregulated in AS versus NC valves.

**Figure 2:**
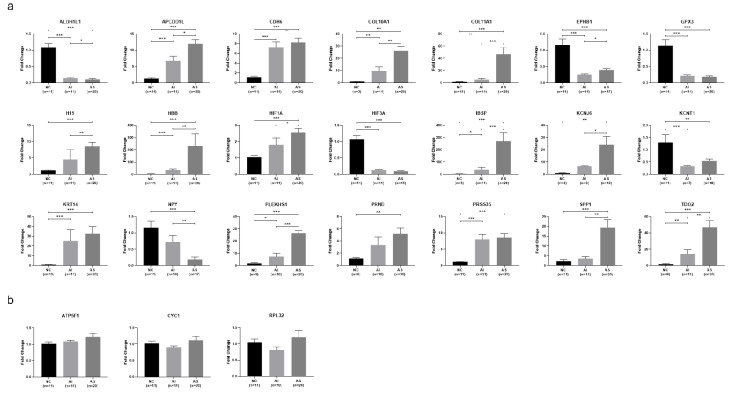
Confirmation of selected differentially expressed genes using quantitative polymerase chain reaction. (**a**) Quantitative polymerase chain reaction (qPCR) was performed for the differentially expressed genes (DEGs) with the greatest expression differences between valves with aortic stenosis (AS) or aortic insufficiency (AI) compared to the normal controls (NC), as well as for selected genes which were identified as DEGs in previous studies. Data are presented as mean ± standard error. * indicates *p* < 0.05, ** indicates *p* < 0.01, *** indicates *p* < 0.001. (**b**) Gene expression of selected housekeeping genes *ATP5F1*, *CYC1*, and *RPL32*, showing no difference in expression when comparing AS, AI, and NC valves. Data are presented as mean ± standard error.

Based on qPCR results (Figure 2a), the genes *APCDD1L*, *CDH6*, *COL10A1*, *IBSP*, *KRT14*, *PLEKHS1*, *PRSS35*, and *TDO2* were confirmed to be significantly upregulated in both AS and AI valves compared to NC valves (*p* < 0.05). One gene (*HBB*) which was found to be upregulated only in AS valves using RNA sequencing was found to be upregulated in both AS and AI valves using qPCR (*p* < 0.05). The genes *ALDH1L1*, *EPHB1*, *GPX3*, *HIF3A*, and *KCNT1* were all confirmed by qPCR to be significantly downregulated in both AS and AI valves compared to NC valves (*p* < 0.05). The genes *COL11A1*, *H19*, *HIF1A*, *KCNJ6*, *PRND*, and *SPP1* were all confirmed by qPCR to be significantly upregulated only in AS valves, and *NPY* was confirmed by qPCR to be significantly downregulated only in AS valves (*p* < 0.05).

The gene that was upregulated to the greatest degree for AS versus NC valves was *IBSP* (fold change 176.6, *p* = 3.76 × 10^−5^), followed by *HBB* (fold change 102.3, *p* = 1.65 × 10^−7^) and *COL11A1* (fold change 34.5, *p* = 2.36 × 10^−8^). The genes that were downregulated to the greatest degree for AS versus NC valves were *HIF3A* (fold change −11.6, *p* = 3.66 × 10^−8^) and *ALDH1L1* (fold change −10.6, *p* = 4.72 × 10^−8^). The gene that was upregulated to the greatest degree for AI versus NC valves was *COL10A1* (fold change 6.6, *p* = 2.17 × 10^−16^), followed by *KRT14* (fold change 6.1, *p* = 1.57 × 10^−12^) and *APCDD1L* (fold change 5.8, *p* = 4.63 × 10^−21^). The genes that were downregulated to the greatest degree for AI versus NC valves were *HIF3A* (fold change −8.6, *p* = 2.57 × 10^−35^), *KCNT1* (fold change −7.3, *p* = 6.56 × 10^−23^), and *ALDH1L1* (fold change −7.0, *p* = 3.42 × 10^−27^).

We identified 11 DEGs that were significantly upregulated in AS versus AI valves using both RNA sequencing and qPCR (*APCDD1L*, *COL10A1*, *COL11A1*, *H19*, *HBB*, *HIF1A*, *IBSP*, *KCNJ6*, *PLEKHS1*, *SPP1*, *TDO2*). The most upregulated DEG was *COL11A1* with a fold change of 9.2 (*p* = 3.28 × 10^−6^), followed by *IBSP* (fold change 7.0, *p* = 9.19 × 10^−4^) and *HBB* (fold change 6.8, *p* = 0.003). We also identified one gene that was significantly downregulated in AS versus AI valves using both RNA sequencing and qPCR (*NPY*, fold change −4.0, *p* = 0.007). Five DEGs were expressed at similar levels in AS and AI valves, as confirmed using both RNA sequencing and qPCR (*GPX3*, *HIF3A*, *KCNT1*, *PRND*, *PRSS35*).

qPCR control validation was performed using the housekeeping genes *ATP5F1*, *CYC1*, and *RPL32* as reference genes. All three were found to not be differentially expressed on RNA sequencing and qPCR when comparing the AS, AI, and NC groups (Figure 2b). The correlation between RNA sequencing and qPCR gene expression results are illustrated for all 21 DEGs in Figure S1.

### 3.3. Gene Ontology and KEGG Analysis

GO analysis showed 7862 enriched GO terms between the AS and NC valves, and 7809 enriched GO terms between the AI and NC valves. Among AS and NC valves, 1511 GO terms were significantly enriched (*p* < 0.05), of which 456 were highly statistically significant (*p* < 0.0005). The significant terms for AS were related to cellular immune response, protein binding, extracellular matrix modulation, and cytokine binding. Among AI and NC valves, a total of 1036 GO terms were significantly enriched (*p* < 0.05), of which 186 were extremely statistically significant (*p* < 0.0005). The significant terms for AI were related to cell growth and migration, cell adhesion, and protein binding.

**Figure 3:**
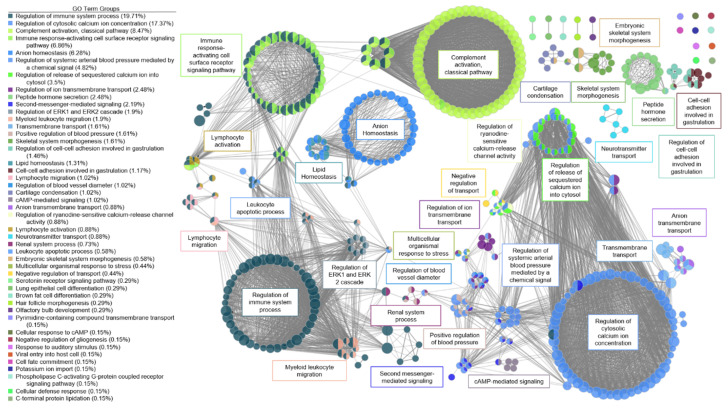
Functional network of genes involved in aortic stenosis. Each node represents a gene ontology (GO) term that has a significant number of differentially expressed genes. Edges represent known associations between GO terms. GO terms are grouped together by similarity and assigned a representative group name. Major clusters for aortic stenosis were ion regulation, immune system response, blood pressure regulation, and lipid homeostasis.

A functional network for AS was created (Figure 3). There were six large clusters and three systems for AS valves. The first system for AS involved ion regulation with several clusters relating to calcium regulation, arterial blood pressure regulation, and second messenger mediated signaling. Via a high-impact bridge of antimicrobial humoral response and renal regulation of blood pressure, the first system was linked to the second system involving immune system response. The major clusters in this second system included regulation of the immune system process, immune response-activating cell surface signaling pathway, and activation of the classical complement pathway. T-cell co-stimulation, lymphocyte co-stimulation, regulation of leukocyte cell-cell adhesion, and positive regulation of T-cell activation were highly connected nodes between the regulation of immune response cluster and the immune response to activating cell surface receptor signaling pathway cluster, with more than 25 connections each. A small cluster of nodes activating the immune response connected the activating cell surface receptor signaling pathway cluster and the classical complement activation cluster, with greater than 45 connections each. The third system for AS was lipid homeostasis. A high-impact bridge through fatty acid regulation and regulation of lipid metabolism connected the lipid homeostasis cluster to the immune regulation system.

**Figure 4:**
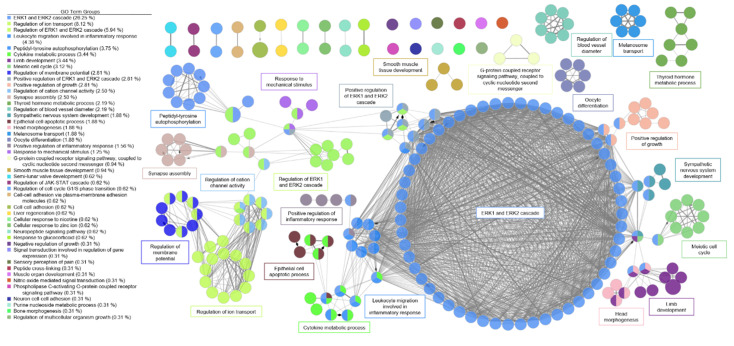
Functional network of genes involved in aortic insufficiency. Each node represents a gene ontology (GO) term that has a significant number of differentially expressed genes. Edges represent known associations between GO terms. GO terms are grouped together by similarity and assigned a representative group name. The major system for aortic insufficiency involved the ERK1 and ERK2 signaling cascade.

A functional network was created for AI in the same manner as described above (Figure 4). For AI, there was one major system with ERK1 and ERK2 regulation as the dominant cluster. Related clusters included immune cell signaling for apoptosis.

KEGG analysis of AS versus NC valves revealed significant enrichment differences in focal adhesion, extracellular matrix receptor interaction, cytokine–cytokine receptor interaction, and cell adhesion (*p* < 0.05). In comparing AI versus NC valves, significant enrichment differences were observed in hypertrophic cardiomyopathy, extracellular matrix receptor interaction, dilated cardiomyopathy, and focal adhesion (*p* < 0.05, Figure S2).

### 3.4. Protein-Protein Interaction Network Analysis

**Figure 5:**
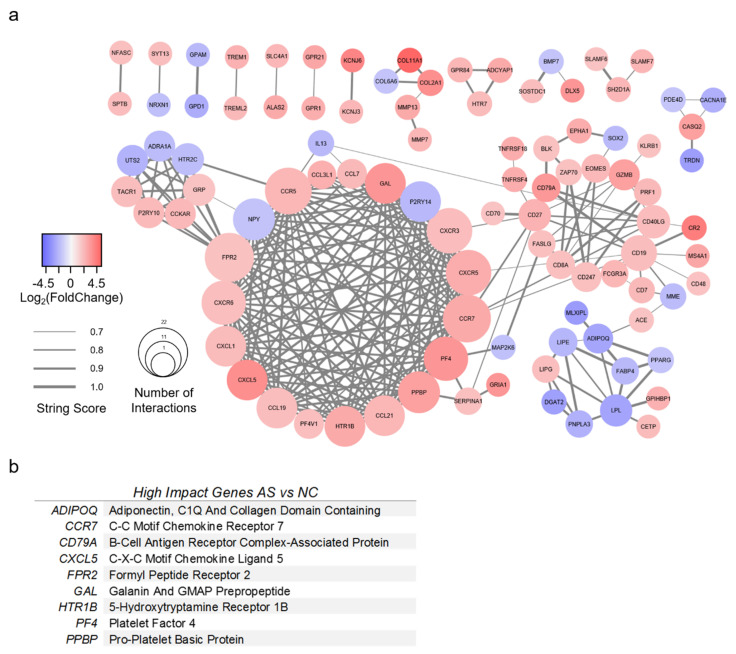
Protein-protein interaction model of valves with aortic stenosis. (**a**) Protein-protein interaction model of differentially expressed genes (DEGs) from RNA sequencing analysis of aortic stenosis (AS) versus normal control (NC) valves. DEGs found in AS versus NC alone with >3-fold change were included. The STRING database was used to generate gene relationships. STRING scores <0.7 were excluded. Edges are weighted to indicate STRING score. A list of high-impact genes was generated from these interactions. (**b**) High-impact genes for AS were defined as having (1) 15 or more interactions; (2) >5-fold absolute change; (3) connectivity to surrounding clusters.

A PPI network was created for AS with 98 genes of interest (Figure 5a). All genes had greater than 3-fold change compared to NC valves, as well as a STRING score of 0.7 or greater. The top nine most impactful genes are listed in Figure 5b. High-impact genes for AS were defined as having (1) 15 or more interactions; (2) >5-fold absolute change; (3) connectivity to surrounding clusters. Genes with greater than 15 interactions were *CCR5*, *CCR7*, *CXCR3*, *CXCR5*, *CXCR6*, *FPR2*, *NPY*, *PF4*, and *PPBP*. The most upregulated genes for AS were *COL2A1*, *COL11A1*, *CR2*, *CXCL5*, *KCNJ6*, and *PF4*. The most downregulated genes for AS were *ADIPOQ*, *DGAT2*, *GPD1*, *LPL*, *MLXIPL*, and *TRDN*. Several genes were identified which bridged gene clusters: *ACE*, *ADIPOQ*, *CCR7*, *CD19*, *CD27*, *CD40LG*, *CD79A*, *FPR2*, *IL13*, *MME*, and *SERPINA1*.

**Figure 6:**
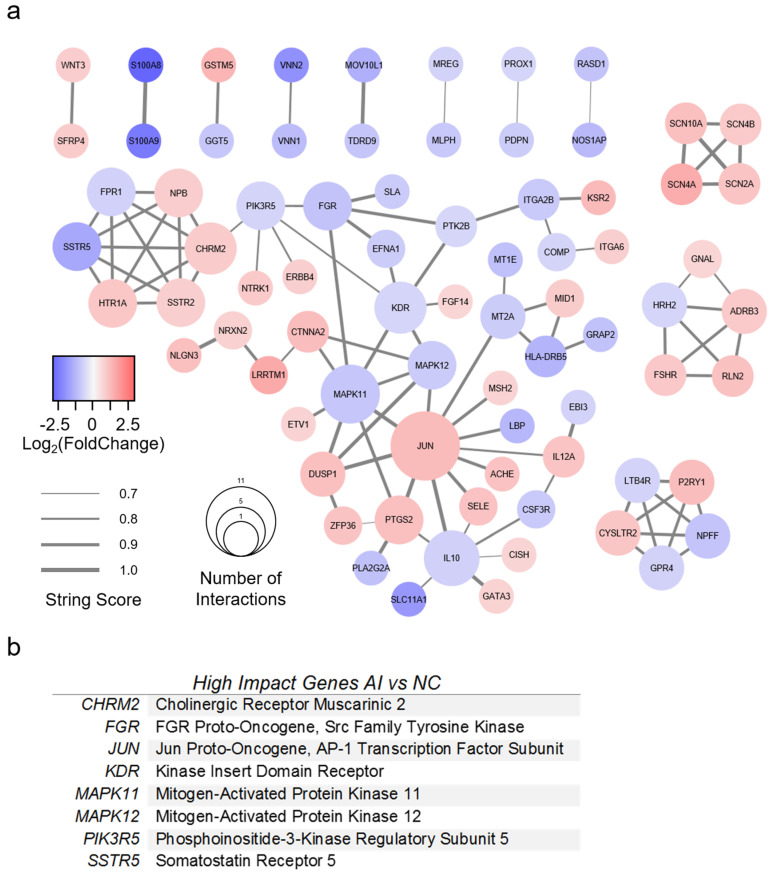
Protein-protein interaction model of valves with aortic insufficiency. (**a**) Protein-protein interaction model of differentially expressed genes (DEGs) from RNA sequencing analysis of aortic insufficiency (AI) versus normal control (NC) valves. DEGs found in AI versus NC alone with >1.5-fold change were included. The STRING database was used to generate gene relationships. STRING scores <0.7 were excluded. Edges are weighted to indicate STRING score. A list of high-impact genes was generated from these interactions. (**b**) High-impact genes for AI were defined as (1) >5 interactions; (2) >2-fold absolute change; (3) connectivity to surrounding cluster.

A PPI network was created for AI with 77 genes of interest (Figure 6a). All genes had greater than 1.5-fold change compared to NC valves, as well as a STRING score of 0.7 or greater. The top eight most impactful genes are listed in Figure 6b. High-impact genes for AI were defined as (1) >5 interactions; (2) >2-fold absolute change; (3) connectivity to surrounding cluster. Genes with greater than five interactions were *CHRM2*, *IL10*, *JUN*, *KDR*, and *MAPK11*. The most upregulated genes (fold change >2) were *CTNNA2*, *GSTM5*, *JUN*, *KSR2*, *LRRTM1*, *P2RY1*, and *SCN4A*. The most downregulated genes were *HLA-DRB5*, *LBP*, *MOV10L1*, *NOS1AP*, *S100A8*, *S100A9*, *SLC11A1*, *SSTR5*, *VNN1*, and *VNN2*. Several genes were identified which bridged gene clusters: *CHRM2*, *CTNNA2*, *FGR*, *ITGA2B*, *JUN*, *KDR*, *MAPK11*, *MAPK12*, *MT2A*, *PIK3R5*, and *PTK2B*.

## 4. Discussion

In this study, we characterized the genetic expression profile of normal, stenotic, and regurgitant human aortic valves to create an updated roadmap for future therapeutic investigations. By identifying potential gene targets, signaling pathways, and protein–protein interactions, our data may help scientists and clinicians not only understand the pathophysiology underlying the chronic, gradual progression of AS and AI, but also develop genetic or pharmacologic interventions to inhibit or even reverse the course of disease. Importantly, our study also represents a step forward from previous transcriptional analyses of human aortic valves, which have all focused on AS [13,14,15,16]. No prior studies have been devoted to examining the gene expression profile of valves with AI, and the existing reports of AS valves compared their gene expression profile against that of aortic valves derived from failing hearts explanted during transplant or from patients with aortic root aneurysms. Thus, our current work includes a potentially more appropriate “normal” control group by using aortic valves from non-matched donor hearts that were otherwise acceptable for transplantation.

We identified 8621 DEGs among the AS, AI, and NC valves in our study, approximately an order of magnitude greater than that of previously published reports. Indeed, among the 499 DEGs identified by Guauque-Olarte et al. between calcified versus non-calcified tri-leaflet aortic valves [13], our study confirmed 439 of these genes (88.0%) to be differentially expressed. Additionally, all 439 of these DEGs matched in the direction of relative expression (i.e., upregulated or downregulated) in both our study and that of Guauque-Olarte et al. This result validates our methodology, confirms the significance of previously published data, and lends credence to our discovering thousands of previously unidentified DEGs. Confirmatory qPCR was additionally performed on the 21 most differentially expressed and promising genes in our study. These included *COL11A1*, *IBSP*, *NPY*, and *SPP1*, which were previously found to be differentially expressed in explanted aortic valves [13]. *HIF1A* was selected based on its involvement in hypoxic states [30,31,32], while *KCNJ6*, *H19*, *PLEKHS1*, *PRND*, and *TDO2* were selected for their high-fold change compared to NC valves.

The general function of many of the upregulated and downregulated DEGs of interest we identified are known, although their roles specifically in the development of AS and AI may not be fully characterized. The most upregulated DEG of interest for AS was *IBSP* (Integrin Binding Sialoprotein), which is involved in calcium regulation and tightly binds hydroxyapatite to function as an integral part of the mineralization matrix [33]. The next most upregulated DEGs of interest involved in AS were *HBB* (Hemoglobin Subunit Beta), which is involved in oxygen transport and oxygen/carbon dioxide exchange [34], and *COL11A1* (Collagen Type XI Alpha 1 Chain), which is involved in extracellular matrix binding and ERK signaling [35]. While *IBSP* and *HBB* were also upregulated in AI, *COL11A1* upregulation was specific to AS. Other important upregulated genes of interest specifically involved with AS included *KCNJ6* (Potassium Voltage-Gated Channel Subfamily J Member 6), and *SPP1* (Secreted Phosphoprotein 1, or Osteopontin), which is involved in osteoclast attachment to the mineralized bone matrix [36]. *NPY* (Neuropeptide Y), which is involved in MAPK signaling, regulation of calcium levels, and activation of potassium channels [37], was the only gene investigated using qPCR found to be downregulated in AS alone. *HIF3A* (Hypoxia Inducible Factor 3 Subunit Alpha), a transcriptional regulator in adaptive responses to low oxygen tension [38], was the most downregulated DEG of interest for AS overall, although *HIF3A* was also the most downregulated DEG for AI as well. For AI, the most upregulated DEG of interest was *COL10A1* (Collagen Type X Alpha 1 Chain), followed by *KRT14* (Keratin 14), which forms the cytoskeleton of epithelial cells [39].

GO and KEGG analysis revealed enrichment of several pathways which have been implicated in the development of aortic valve disease. We created a functional network based on our DEGs that demonstrated potential interactions in the regulation of AS and AI. The most promising molecular pathways for AS involved ion channel dysregulation, immune response, and blood pressure regulation. This functional network illustrates the complex interaction between the inflammatory state, calcium regulation, and secondary messenger cascades which have also been observed in the clinical setting [40]. For AI, the most dominant signaling pathways were the ERK1 and ERK2 pathways. A host of secondary messenger molecules were also implicated in the functional network for AI.

Finally, we generated PPI networks that identified high-impact genes involved in the regulation of AS and AI. For example, *ADIPOQ* (Adiponectin) was found to be a high-impact gene downregulated in the AS PPI network. Decreased levels of adiponectin have been implicated in the pathogenesis of AS [41], and adiponectin is known to be a collagen-like plasma protein that inhibits TNF-α, thereby decreasing inflammation [42,43]. Genetic modulation of *ADIPOQ* in patients with mild or moderate AS may potentially be beneficial, perhaps by slowing, stopping, or even reversing disease progression. We have identified numerous other potential gene targets, each of which may be explored further for potential therapeutic application.

Our study is subject to several important limitations. First, our NC valves were derived from unmatched donor hearts that were acceptable for transplantation. As a result, the age of the donors who contributed the NC valve tissue were not matched to the age of our patients with AS and AI. This discrepancy is difficult to remedy, as heart donors are typically young, while patients with aortic valve disease are generally older, as the disease processes take considerable time to become severe enough to warrant surgical intervention. As some transcriptional changes may occur in aortic valve tissues due to the natural aging process, not all of the DEGs we identified may actually represent differences between AS or AI versus NC. However, our use of NC valves from donor hearts is also a significant strength of our study, as previous reports used valves from transplant recipients’ failing hearts or from patients with aortic root aneurysms as a control, in which confounding gene expression pathways activated in the setting of cardiomyopathic or aortopathic processes may distort their results. Although donor hearts cannot be considered truly healthy, the NC valves used in our study nevertheless offer a new perspective of “normal” in the absence of significant cardiac disease, thereby representing a potentially more appropriate control group.

Another limitation of our study lies in the fact that the valves used in our study generally represented late-stage disease. Although AS and AI represent chronic disease processes with continuous progression over time, such that mechanistic gene expression changes may remain active even during the later stages, some of the DEGs we identified may not reflect the mechanism of aortic valve disease development but instead characterize the transcriptome of end-stage AS or AI. In addition, our small sample size of only five valves per group may lead to false positive and false negative signals due to chance. Furthermore, because we processed the entire aortic valve leaflet in our samples, our gene expression analysis represents the composite differential gene expression of all cell types within the aortic valve leaflet. Future single-cell RNA sequencing experiments may be performed to parse the transcriptional differences within particular cell types found within normal and diseased aortic valve leaflets.

Finally, our AS and AI patients unavoidably presented with other cardiovascular and systemic medical conditions, commonly including coronary artery disease, diabetes, hypertension, and arrhythmias. Although we were able to exclude patients with systemic infections, inflammatory conditions, and connective tissue disorders, we acknowledge that the gene expression profiles obtained for the AS and AI groups do not represent aortic valve disease in isolation. Nevertheless, as the goal of our study is to establish targets for the development of novel translational therapies, it is important to consider that our data is a clinically relevant representation of real patients who presented with AS and AI. Future therapies aimed at genetically or molecularly altering the course of AS and AI will be done in patients with similar comorbidities.

## 5. Conclusions

Overall, we sequenced aortic valve leaflets from non-matched transplant donor hearts, thereby establishing a new standard for normal aortic valve transcriptional profiling. We also sequenced human aortic valves with AI and demonstrated that valves with AI and AS possess unique gene expression patterns. The plethora of DEGs identified and our novel PPI networks for AS and AI have revealed new pathways missed by previous aortic valve sequencing studies. Our data lays the foundation for future gene editing studies and may guide researchers in developing novel therapeutic interventions for AS and AI.

## Figures and Tables

**Table 1:** Baseline patient characteristics.

Group	Age (Years)	Sex	BMI (kg/m^2^)	Aortic Valve	Other Medical History	LVEF
AS-1	71	F	24.7	Severe AS	Bicuspid aortic valve, coronary disease, hypertension	56.0%
AS-2	74	F	33.0	Severe AS	Hypertension	60.0%
AS-3	79	M	29.8	Severe AS	Coronary disease, diabetes, hypertension, sick sinus syndrome	59.0%
AS-4	73	M	25.7	Severe AS	Heart failure, moderate mitral valve regurgitation, severe tricuspid valve regurgitation, hypertension, atrial fibrillation	26.0%
AS-5	72	F	33.2	Severe AS	Prior coronary bypass surgery, diabetes, hypertension, atrial fibrillation	62.5%
AI-1	71	M	30.9	Moderate AI	Coronary disease, hypertension, atrial fibrillation	57.0%
AI-2	63	M	35.3	Severe AI	A 5.3 cm dilated aortic root, heart failure, coronary disease, hypertension, atrial fibrillation	34.0%
AI-3	79	M	24.1	Severe AI	Heart failure, severe mitral and tricuspid valve regurgitation, coronary disease, hypertension	56.0%
AI-4	77	M	29.0	Severe AI	Hypertension, atrial fibrillation	56.0%
AI-5	55	F	23.0	Moderate AI	Radiation-induced heart disease, severe mitral valve regurgitation, coronary disease	62.0%
NC-1	56	F	22.0	Normal	Ruptured internal carotid artery aneurysm, diabetes, hypertension	75.0%
NC-2	17	F	21.6	Normal	Anoxic brain injury	62.0%
NC-3	31	M	36.0	Normal	Anoxic brain injury, diabetes, hypertension, intravenous drug use	65.0%
NC-4	21	F	25.0	Normal	Tonsillar herniation after drug overdose	48.0%
NC-5	61	F	20.4	Normal	Intracranial hemorrhage	67.0%

A total of 15 patients were included in the study, with five patients in each of the normal control (NC), aortic stenosis (AS), and aortic insufficiency (AI) groups. BMI, body mass index; F, female; LVEF, left ventricular ejection fraction; M, male.

**Table 2:** Results of RNA sequencing and quantitative polymerase chain reaction.

		*AS vs. NC*				*AI vs. NC*				*AS vs. AI*			
		RNA-seq		qPCR		RNA-seq		qPCR		RNA-seq		qPCR	
*Gene*		FC	*p*-Adj	FC	*p*-Value	FC	*p*-Adj	FC	*p*-Value	FC	*p*-Adj	FC	*p*-Value
*ALDH1L1*	Aldehyde Dehydrogenase 1 Family Member L1	−12.15	4.61 × 10^−49^	−10.59	4.72 × 10^−8^	−7.00	3.415 × 10^−27^	−7.88	2.84 × 10^−6^	−1.31	2.98 × 10^−1^	−1.34	2.9 × 10^−2^
*APCDD1L*	Adenomatosis Polyposis Coli Down-Regulated 1 Protein-Like	17.31	5.65 × 10^−80^	9.41	1.65 × 10^−7^	5.81	4.626 × 10^−20^	5.36	1.98 × 10^−5^	2.01	2.82 × 10^−4^	1.76	1.4 × 10^−2^
*CDH6*	Cadherin 6	10.18	5.66 × 10^−34^	7.26	2.36 × 10^−8^	5.36	3.201 × 10^−38^	6.37	5.67 × 10^−6^	1.76	2 × 10^−3^	1.14	6.70 × 10^−1^
*COL10A1*	Collagen Type X Alpha 1 Chain	37.65	1.56 × 10^−40^	25.03	1.13 × 10^−3^	6.56	2.168 × 10^−16^	9.35	1 × 10^−2^	3.56	1.46 × 10^−12^	2.68	2 × 10^−3^
*COL11A1*	Collagen Type XI Alpha 1 Chain	23.01	4.42 × 10^−59^	34.48	2.36 × 10^−8^	−1.22	5.46 × 10^−1^	3.75	2.4 × 10^−1^	12.18	4.30 × 10^−30^	9.20	3.28 × 10^−6^
*EPHB1*	Ephrin Type-B Receptor 1	−6.01	2.24 × 10^−32^	−2.94	8.28 × 10^−5^	−5.99	4.948 × 10^−31^	−4.57	1.13 × 10^−5^	1.14	6.92 × 10^−1^	1.56	1.3 × 10^−2^
*GPX3*	Glutathione Peroxidase 3	−6.49	1.37 × 10^−35^	−6.12	9.45 × 10^−8^	−6.23	5.968 × 10^−56^	−5.35	2.84 × 10^−6^	1.00	9.90 × 10^−1^	−1.14	1.69 × 10^−1^
*H19*	H19 Imprinted Maternally Expressed Transcript	12.63	4.72 × 10^−41^	7.42	2.83 × 10^−7^	1.10	8.06 × 10^−1^	3.97	3 × 10^−1^	6.08	1.41 × 10^−15^	1.87	2 × 10^−3^
*HBB*	Hemoglobin Subunit Beta	27.27	6.07 × 10^−31^	102.27	1.65 × 10^−7^	4.97	1.456 × 10^−11^	15.09	5.398 × 10^−5^	3.48	1.19 × 10^−10^	6.78	3 × 10^−3^
*HIF1A*	Hypoxia Inducible Factor 1 Subunit Alpha	1.66	1.00 × 10^−4^	2.44	4.61 × 10^−6^	1.05	7.51 × 10^−1^	1.73	1.2 × 10^−1^	1.54	3 × 10^−3^	1.41	4.4 × 10^−2^
*HIF3A*	Hypoxia Inducible Factor 3 Subunit Alpha	−17.22	3.90 × 10^−77^	−11.58	3.66 × 10^−8^	−8.61	2.574 × 10^−35^	−8.74	2.84 × 10^−6^	−1.44	1.66 × 10^−1^	−1.32	1.58 × 10^−1^
*IBSP*	Integrin Binding Sialoprotein	32.09	3.23 × 10^−19^	176.57	3.76 × 10^−5^	2.74	2.51 × 10^−4^	25.19	1.39 × 10^−2^	8.85	6.01 × 10^−20^	7.01	9.19 × 10^−4^
*KCNJ6*	Potassium Voltage-Gated Channel Subfamily J Member 6	11.61	7.62 × 10^−10^	23.18	9.52 × 10^−3^	−1.04	NA	6.36	2 × 10^−1^	4.72	1.74 × 10^−9^	3.65	3.0 × 10^−2^
*KCNT1*	Potassium Sodium-Activated Channel Subfamily T Member 1	−4.91	2.14 × 10^−8^	−2.40	9.39 × 10^−3^	−7.32	6.56 × 10^−23^	−3.94	2 × 10^−3^	1.53	2.19 × 10^−1^	1.64	8.5 × 10^−2^
*KRT14*	Keratin 14	38.09	7.93 × 10^−24^	31.05	2.66 × 10^−7^	6.10	1.57 × 10^−12^	24.01	5.67 × 10^−6^	2.19	1 × 10^−3^	1.29	2.98 × 10^−1^
*NPY*	Neuropeptide Y	−3.14	1.7 × 10^−2^	−6.37	9.03 × 10^−6^	1.26	5.28 × 10^−1^	−1.61	2 × 10^−1^	−3.41	4.10 × 10^−6^	−3.97	7 × 10^−3^
*PLEKHS1*	Pleckstrin Homology Domain Containing S1	60.77	8.91 × 10^−52^	14.72	2.00 × 10^−7^	2.14	7 × 10^−3^	4.10	1.3 × 10^−2^	7.11	1.62 × 10^−18^	3.59	1.79 × 10^−5^
*PRND*	Prion Like Protein Doppel	77.29	9.16 × 10^−44^	4.66	3.37 × 10^−3^	1.35	1.70 × 10^−1^	2.99	8.7 × 10^−1^	1.30	4.86 × 10^−1^	1.56	1.0 × 10^−1^
*PRSS35*	Serine Protease 35	13.41	1.00 × 10^−35^	7.88	1.65 × 10^−7^	5.48	2.991 × 10^−15^	7.34	2.84 × 10^−6^	1.49	2.02 × 10^−1^	1.07	7.92 × 10^−1^
*SPP1*	Secreted Phosphoprotein 1	17.49	1.67 × 10^−23^	8.49	4.65 × 10^−5^	1.90	1.2 × 10^−2^	1.51	2.5 × 10^−1^	5.81	5.22 × 10^−15^	5.61	7.54 × 10^−4^
*TDO2*	Tryptophan 2,3-Dioxygenase	24.73	3.80 × 10^−19^	27.71	6.43 × 10^−7^	2.12	7 × 10^−3^	8.37	4 × 10^−3^	3.44	3.10 × 10^−6^	3.31	2 × 10^−3^
*ATP5F1*	ATP Synthase Peripheral Stalk-Membrane Subunit B	−1.03	7.43 × 10^−1^	1.20	2.98 × 10^−1^	−1.05	5.21 × 10^−1^	1.07	1.7 × 10^−1^	1.01	9.11 × 10^−1^	1.13	8.55 × 10^−1^
*CYC1*	Cytochrome C1	1.00	9.97 × 10^−1^	1.09	7.37 × 10^−1^	−1.05	6.20 × 10^−1^	−1.13	2.2 × 10^−1^	1.05	8.32 × 10^−1^	1.24	4.27 × 10^−1^
*RPL32*	Ribosomal Protein L32	1.07	7.61 × 10^−1^	1.15	8.87 × 10^−1^	1.00	9.75 × 10^−1^	−1.30	2.5 × 10^−1^	1.05	8.48 × 10^−1^	1.49	3.28 × 10^−1^

Gene expression of 21 differentially expressed genes and three housekeeping genes were obtained from valves with aortic stenosis (AS), aortic insufficiency (AI), and normal controls (NC) using RNA sequencing (RNA-seq) and quantitative polymerase chain reaction (qPCR). Fold changes (FC) in gene expression and adjusted *p*-values (*p*-adj) are listed, comparing AS vs. NC, AI vs. NC, and AS vs. AI. For *KCNJ6* in the AI group, no reads were mapped to the gene locus.

## Supplementary Materials

The following are available online at https://www.mdpi.com/2073-4425/11/7/789/s1, Figure S1: Comparison of RNA sequencing and quantitative polymerase chain reaction results; Figure S2, Kyoto Encyclopedia of Genes and Genome enrichment analysis.
