# Supplementary material for: Transcriptional Profiling of Normal, Stenotic, and Regurgitant Human Aortic Valves
Source: Genes (Basel). 2020 Jul 14;11(7):789. doi: 10.3390/genes11070789 (PMC7397246; doi:10.3390/genes11070789)
Supplement: Supplementary file 1 [file genes-11-00789-s001.pdf]

a

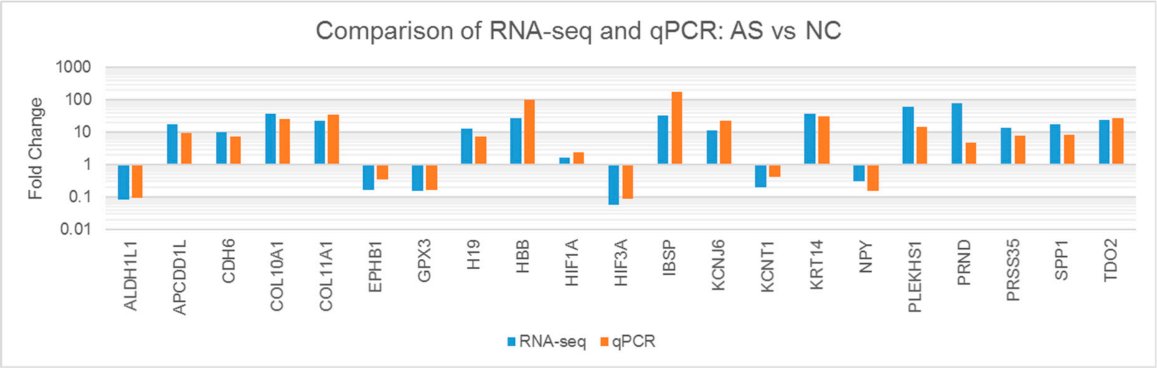

b

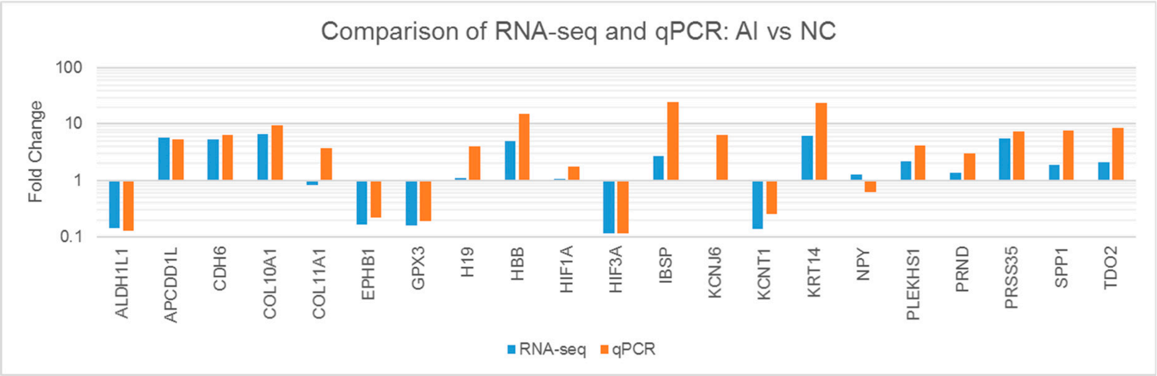

Figure S1: Comparison of RNA Sequencing and Quantitative Polymerase Chain Reaction Results

RNA sequencing (RNA-seq) and quantitative polymerase chain reaction (qPCR) results are expressed as fold changes in gene expression for 21 differentially-expressed genes. A fold change of 1 indicates no differential expression. Results for aortic stenosis (AS) versus normal controls (NC) are shown in **(a)**, and results for aortic insufficiency (AI) versus NC are shown in **(b)**.

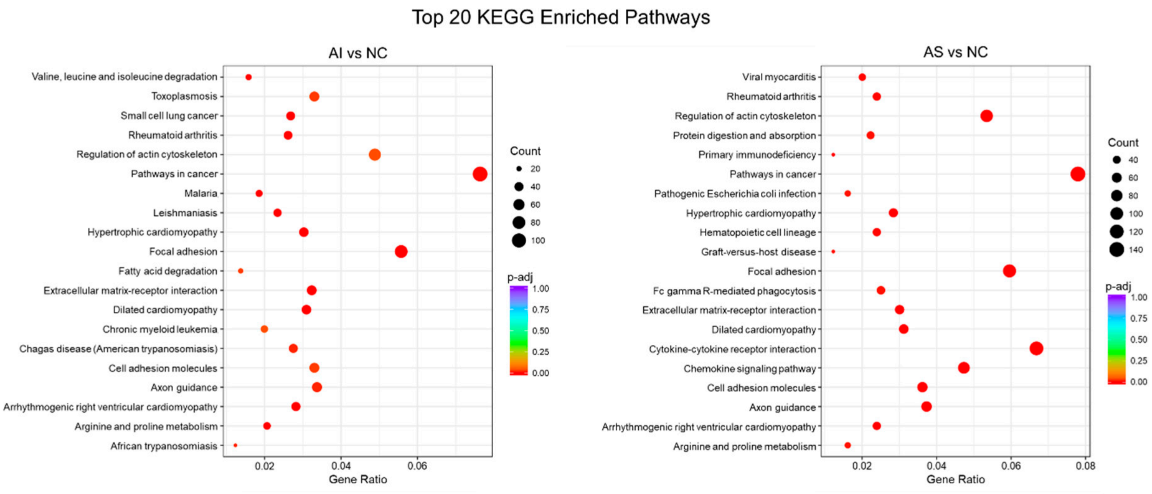

Figure S2: Kyoto Encyclopedia of Genes and Genome Enrichment Analysis

Kyoto Encyclopedia of Genes and Genome (KEGG) enrichment analysis was performed. The top 20 most enriched pathways for aortic stenosis (AS) or aortic insufficiency (AI) versus normal control (NC) valves are shown.
